# Parametric Evaluation of the CFK and the MIL-STD-1472 Equations as Tools for the Estimation of Blood COHb Levels

**DOI:** 10.3390/toxics14060488

**Published:** 2026-06-03

**Authors:** Jacob Alter, Avraham Dayan, Gideon Fleminger

**Affiliations:** The Shmunis School of Biomedicine and Cancer Research, George S. Wise Faculty of Life Sciences, Tel Aviv University, Tel Aviv 6997801, Israel

**Keywords:** carbon monoxide exposure, carboxyhemoglobin, CO toxicity, armored motor vehicles, MIL-STD-1472, CFK equation

## Abstract

Carbon monoxide (CO) is a colorless, odorless byproduct of incomplete combustion that binds to hemoglobin to form carboxyhemoglobin (COHb), impairing oxygen delivery and causing systemic hypoxia. Two widely used models for estimating CO toxicity are the Coburn–Forster–Kane (CFK) equation, which incorporates physiological and anthropometric parameters, and the workload-based MIL-STD-1472 model, developed in the late 1960s and 1970s. Both have been applied extensively in military armored motor vehicle (AMV) operations, firefighting, and mining. This study evaluates the predictive performance of these models during field trials involving AMV crews conducting live-fire exercises. Ambient CO concentrations were continuously monitored, and serial blood samples were obtained for COHb determination. Individual physiological and anthropometric data were used to generate CFK-based predictions, while the MIL-STD-1472 estimates were derived using the activity level equivalent (ALE) values, which were developed to allow for mathematical alignment between the two models. Measured COHb levels showed strong agreement with predictions from both the CFK and MIL-STD-1472 equations. An ALE analysis indicated that the actual physical workload during AMV operations was substantially lower than the activity level (AL = 4) originally recommended in MIL-STD-1472. In addition, algebraic rearrangement of the MIL-STD-1472 equation enabled the estimation of COHb recovery time following cessation of exposure. This study provides a field-based evaluation of two established models for predicting carboxyhemoglobin formation. Measurements obtained during live armored motor vehicle operations demonstrate that both the CFK and MIL-STD-1472 equations accurately estimate COHb under conditions of near-ambient oxygen tension and minimal CO_2_ accumulation. Importantly, MIL-STD-1472 predictions using moderate ALE values were more consistent with the observed COHb values, suggesting that the commonly applied activity level (AL = 4) may overestimate CO exposure risk in comparable operational environments.

## 1. Introduction

### 1.1. Carbon Monoxide (CO) Toxicity

Carbon monoxide is a colorless, odorless, and tasteless gas generated during the incomplete combustion of carbonaceous compounds, particularly in poorly ventilated environments. CO poisoning remains a major public health concern, accounting for more than 50,000 emergency department visits annually in the United States alone [1,2]. Comprehensive reviews of CO toxicity and pathophysiology are available elsewhere [1,2,3,4,5,6]. Exposure to CO arises from environmental sources, such as vehicle exhaust and domestic heating systems, as well as occupational settings, including mining, firefighting, and industrial work. During military activities, soldiers performing weapons firing in confined or semi-enclosed platforms may experience acute CO exposure during both training and combat operations [7].

Unlike many inhaled toxicants, CO is not effectively removed by conventional respiratory protective equipment [8], complicating exposure mitigation. Specific metal-activated resins that are capable of adsorbing CO have been developed [9]. CO catalytic conversion into the less toxic CO_2_ usually requires high temperatures [10]. Recently, we developed a Ti-Pt catalyst that effectively reduces toxicity levels at room temperature by the conversion of CO into CO_2_ [11]. Still, exposure remains a concern in operational settings where rapid CO accumulation may occur.

CO is also produced endogenously through the oxidative degradation of heme prosthetic groups in hemoproteins, which takes place mainly in the liver, spleen, brain, and the hemopoietic system by heme oxygenases (HOs), HO1, in particular [12,13,14]. The activity of these enzymes is increased in response to oxidative stress [15,16] and, in certain hemolytic diseases [17], leads to increased basal levels of blood COHb (COHb_0_).

The pathophysiology of CO toxicity is primarily mediated by its high affinity for hemoproteins in the body. The binding of CO to hemoglobin (Hb) forms COHb, reducing the oxygen-carrying capacity and shifting the oxyhemoglobin dissociation curve to the left (a phenomenon known as the Haldane effect), thereby impairing oxygen delivery to tissues [18,19]. CO also binds to intracellular metalloproteins, such as myoglobin and cytochrome oxidases, disrupting mitochondrial respiration and contributing to cellular hypoxia. These effects, which impair oxygen transfer to body tissues, may lead to long-term damage to high-oxygen-demanding organs, such as the lung, liver, kidneys, and brain [20,21,22]. These effects may lead to increased rates of myocardial infarction, pulmonary dysfunction, and strokes, as demonstrated among firefighters upon long-term exposure to fire-generated CO [23].

Acute carbon monoxide (CO) poisoning represents a critical medical emergency characterized by an intricate cascade of tissue hypoxia, inflammatory signaling, and mitochondrial dysfunction [6,24]. Consequently, patients face an elevated risk of severe immediate and chronic neurological sequelae, with damage heavily localized to regions such as the globus pallidus and subcortical white matter [6,25]. Beyond acute lesions, up to 30% of severe poisoning cases culminate in delayed neuropsychiatric syndrome (DNS), where patients experience an abrupt onset of cognitive regression, motor abnormalities, and psychiatric deterioration [25] following a misleading “lucid interval” of up to several weeks [26,27]. The pathophysiology of DNS is uniquely complex, driven by an ischemia-reperfusion injury cascade that triggers lipid peroxidation, microglial activation, and progressive white matter demyelination [28]. While timely administration of hyperbaric oxygen therapy (HBOT) can accelerate COHb clearance and attenuate long-term neurological damage, a substantial percentage of survivors remain burdened with permanent neurocognitive disabilities [29]. Modeling the precise real-time kinetics of COHb generation is therefore essential to mapping exposure windows and optimizing neuroprotective clinical interventions.

### 1.2. Carboxyhemoglobin Generation Models

The severity of CO toxicity depends on both the ambient concentration of the gas and the exposure duration. Increasing COHb levels are associated with a graded spectrum of clinical effects, ranging from headache and dizziness to loss of consciousness, “functional anemia” [30,31], and even death [32]. Accordingly, regulatory agencies have established occupational exposure limits, typically ranging from 25–50 ppm for time-weighted exposure, with concentrations exceeding 1200 ppm classified as immediately dangerous to life and health [33,34]. However, these limits are poorly suited to military environments, such as AMVs [35] and submarines [36], where exposure is characterized by brief, high-intensity CO spikes rather than steady-state conditions. These exposures differ significantly from occupational scenarios, necessitating alternative risk assessment strategies. In such cases, CO toxicity is estimated by calculating the blood COHb concentrations. To address this challenge, mathematical models have been developed to estimate the blood COHb concentrations from ambient CO measurements. One of the most useful models is the Coburn–Forster–Kane (CFK) equation [37], which incorporates individual physiological and pulmonary parameters. It has demonstrated strong empirical support in healthy populations and has been shown to provide the most accurate predictions under controlled conditions, although its performance may vary in specific or vulnerable populations [38,39,40,41,42,43,44]. The CFK equation can be expressed as follows:

(1) $$\frac{d\left[\mathrm{COHb}\right]}{dt}=\left(\frac{{\dot{V}}_{\mathrm{CO}}}{V_{b}}+\frac{1}{V_{b}\cdot b}\right)\left(P_{i\mathrm{CO}}\frac{\left[\mathrm{COHb}\right]\cdot P_{iO_{2}}}{\left[{\mathrm{HbO}}_{2}\right]\cdot M}\right)$$

or, in its integrated form, as follows:

(2) $${COHb}_{t}={COHb}_{0}{\cdot e}^{-\frac{t}{A}}+M\left(1-e^{-\frac{t}{A}}\right)\left({\dot{V}}_{CO}\cdot b+P_{iCO}\right)$$

where *M* is the relative affinity of hemoglobin for CO versus O_2_, ${\dot{V}}_{CO}$ is the endogenous CO production, and $P_{iCO}\;$ and $P_{iO_{2}}$ are the inspired partial pressures of CO and O_2_, respectively.

The parameters ***b*** and ***A*** are defined as follows:

(3) $$b=\frac{1}{DLco}+\frac{\left(P_{A}-P_{H_{2}O}\right)}{{\dot{V}}_{a}}$$

(4) $$A=\frac{M\cdot [{HbO}_{2}]}{P_{aO2}}\cdot V_{b}\cdot b$$

where DLco is the lung diffusion capacity for CO and ${\dot{V}}_{a}$ is the alveolar ventilation rate. Recently, several attempts have been made to review and to re-establish the factors that are associated with the CFK equation [45,46,47]. In 2013, Coburn reexamined some parameters of the Halden and the CFK equations and concluded that the results supported the validity of the CFK model [43].

For military applications, the CFK model was adapted into the MIL-STD-1472 framework [48], which replaces individual physiological parameters with population-averaged parameters and expresses pulmonary gas exchange as a function of activity level (AL), as follows [36,49]:

(5) $${COHb}_{t+\Delta t}={COHb}_{t}\cdot e^{-\frac{\Delta t}{A}}+218\left(1-e^{-\frac{\Delta t}{A}}\right)\cdot \;\left(\frac{1}{B}+\frac{\left[CO\right]}{1403}\right)$$

where ***A*** and ***B*** are AL-dependent constants and $\left[CO\right]$ is the ambient CO concentration in ppm. Thus, in contrast to the CFK model, the MIL-STD-1472 allows for the prediction of COHb generation according to the AL value rather than the determination of physiological and anthropometric parameters of the individual subjects.

Note that the ***b*** factor in the CFK equation was replaced by

***B*** = 1/(V_CO_ · ***b***) (6)

In contrast to the CFK model, the MIL-STD-1472 assumes COHb_0_ = 1%, an assumption that restricts the application of this model to non-smoking subjects, as smokers possess much higher basal COHb concentrations in their blood [50,51]. As shown in Table 1, the ***A***, ***b***, and **B** factors strongly depend on the AL. The correlation between the ***b*** factor and the AL is used to calculate the ALE. The ALE enabled us to apply AL values on a decimal scale, which are determined individually for each participant as follows:

ALE = 0.2459/***b*** − 0.4018 (7)

Despite its age, the continued use of the CO exposure model embedded in the MIL-STD-1472, which is fundamentally based on the classical CFK model, can be explained by a combination of physiological validity, practical simplicity, regulatory inertia, and operational utility. It is widely accepted as the gold standard. Several other mathematical and empirical models are used across toxicology, occupational health, and computational biology to predict carboxyhemoglobin (COHb) generation. There are several examples, including the Peterson–Stewart model [53], the Forbes equation [54], and the Bruce model [55]. These alternative models typically strike a balance between structural simplicity (for fast field assessments) and complex physiological precision (accounting for gas diffusion and non-equilibrium states).

Given their widespread use, including by the Israel Defense Forces (IDF), the CFK and MIL-STD-1472 models are central to decisions regarding allowable firing rates, ammunition type, and ventilation strategies. These models are also being used for civilian purposes, such as by firefighters [56,57] and miners’ security [58,59]. Nonetheless, in 2008, a report by a Review Committee of the American National Research Council (NRC) concluded that experimental data validating CFK-based models under rapidly fluctuating CO exposures typical of AMV cabins were lacking and specifically recommended field validation using real-time CO measurements [7]. The NRC comments motivated, in part, the current project.

In this context, the present study evaluates the predictive performance of both the CFK and MIL-STD-1472 models in estimating COHb accumulation among AMV crews during live-fire exercises. Using continuous CO monitoring and serial blood sampling, we assessed the model accuracy under realistic operational conditions and introduced the concept of the ALE to enable a quantitative alignment between physiological and AL-based modeling approaches. Obviously, in contrast to the AL integer scale, the ALE values are on a decimal scale.

## 2. Materials and Methods

### 2.1. Participants

Field experiments were conducted in fully enclosed AMVs. A total of one hundred male crew members (age 18–22 yr) volunteered to participate and were exposed to carbon monoxide (CO) generated by controlled gunfire during training exercises. One hundred participants enrolled. Before enrollment, all participants provided written informed consent and completed a standardized questionnaire documenting age, height, weight, smoking status, and habitual physical activity. The study protocol was reviewed and approved by the Review Board (Helsinki Committee) of the IDF Medical Corps. Of the 100 participants, 10 either withdrew from the experiment or were excluded for technical reasons (e.g., malfunction of the equipment).

### 2.2. Experimental Design and Firing Protocols

**Figure 1:**
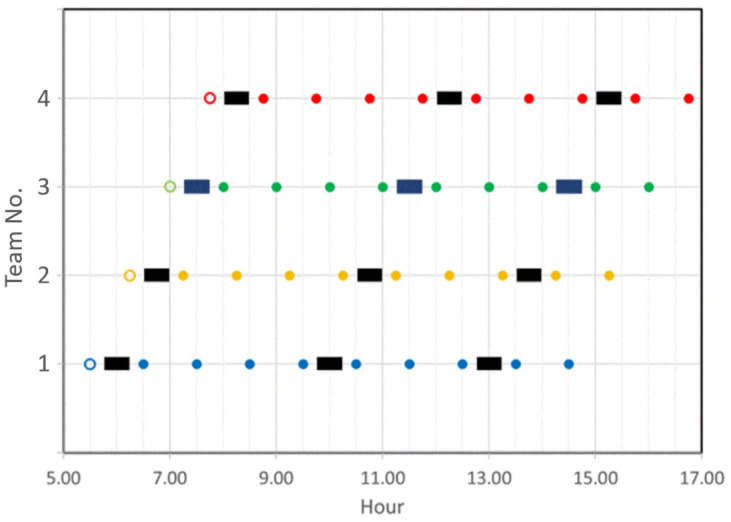
Timetable for firing and blood collection from four teams in a single vehicle during Session 3. Circles represent blood sample collections. Rectangles represent firing periods and CO monitoring. Open circles represent pre-fire blood sampling, samples that were used for the determination of COHb_0_ concentrations.

Three distinct firing scenarios were conducted on separate days and at various locations. Each scenario involved multiple firing sequences separated by vehicle ventilation periods to reduce any residual CO accumulation. A representative timeline of the firing events, CO monitoring, and blood sampling for Session 3 is shown in Figure 1.

It is pertinent to note that, during the intermissions between firing Sessions, the participants were engaged in low-activity instructional tasks.

### 2.3. CO Monitoring

Ambient CO concentrations were monitored using 0–2000 ppm range Gas-Pro electrochemical detectors (Crowcon Detection Instruments Ltd., Abingdon, UK) located at the team positions and serially connected to data loggers, sampled at a 0.1 Hz sampling rate. The detectors were calibrated before each experiment using standard 100 and 500 ppm CO cans (Emproco Ltd, Ashkelon, Israel).

### 2.4. Blood Sampling

Venous blood samples (2 mL) were collected at hourly intervals from all participants (Figure 1) into EDTA-containing Vacutainer tubes (Becton Dickinson and Company, Franklin Lakes, NJ, USA) by certified nursing personnel in a nearby dispensary. During Session 1, an additional sample was collected inside the vehicle during the gun-firing by an onboard physician. The blood samples were transported in ice boxes to an accredited hospital laboratory for COHb analysis by CO-oximetry (cobas b 221 system, Roche Diagnostics Ltd., Rotkreuz, Switzerland). The CO-oximeter was calibrated daily with standard calibration solutions (Roche Diagnostics Ltd., Rotkreuz, Switzerland) and the inter-assay variability was assessed. An analysis was performed within four hours of collection, a time period during which ex vivo COHb blood concentrations remain stable [60,61]. Baseline COHb concentrations (COHb_0_) were defined as those measured in pre-exposure samples collected immediately before firing.

### 2.5. Determination of Physiological Parameters

#### 2.5.1. CFK Equation

The individual physiological parameters required by the CFK equation were determined as follows: the barometric pressure was calculated from the elevation above sea level [62]; tidal volumes (Vt) were determined using a portable spirometer (Vitalograph Ltd., Buckinghamshire, UK); and the DLco was determined using the single-breath technique [63]. Other parameters were calculated as described in Table 2, and Blood oxygen concentrations were measured by pulse oximetry (Masimo, Irvine, CA, USA). COHb_0_ is the COHb concentration measured in the first blood sample of each subject, withdrawn before gunfire began. All other parameters were substituted with the following population-average values: M = 218, Vco = 0.007 mL/min, P_H2O_ = 47, Pi_O2_ = 100 mm Hg, and Pi_CO_ = [CO]/1403. The use of population-average values for selected physiological constants follows the original MIL-STD-1472 framework and reflects the standard operational implementation of the model [48].

Because the total duration of the experiment spanned 10 h, of which 8.5 h were spent in stationary, non-firing rest conditions, the time-weighted average physiological state of the subjects was heavily dominated by the rest period.

#### 2.5.2. The MIL-STD-1472 Model and the ALE

The ***B*** factors were calculated from the ***b*** factors using Equation (6). The ***A*** factors are the same as in the CFK equation. Since COHb_0_ = 1% is assumed by the MIL-STD-1472, the application of this model is limited to non-smoking subjects, as smokers possess much higher basal COHb concentrations in their blood [50,51].

As mentioned above, to enable a quantitative comparison between the CFK-derived physiological parameters and the MIL-STD-1472 activity levels, an ALE value was calculated for each ***b*** factor according to Equation (7).

### 2.6. COHb Recovery Kinetics

Rearrangement of the MIL-STD-1472 equation allows for the calculation of two important time constants, which are highly important in military applications. One is the elapsed time estimated for reaching the maximal allowed COHb concentration (usually 10%) at a given CO concentration and a certain AL:

(8) $$t↑=-A\;ln[1\;-\frac{\left(COHbmax-COHb\left(t\right)\right)}{\left(\frac{218}{B}+\frac{218}{1403}\cdot [CO]\;-\;COHb\left(t\right)\right)}]$$

Similarly, when CO is eliminated from the environment, the time required for returning to COHb_0_ is calculated:

(9) $$t↓=-A\;ln[1\;-\frac{\left(COHb\left(t\right)-\;COHb(0)\right)}{\left(COHb\left(t\right)-\frac{218}{B}\;\right)}]$$

Accordingly, the half-life time values for the reduction of COHb can be calculated:

(10) $$t½=-A\;ln[1\;-0.5\frac{COHbmax}{\left(COHbmax-\frac{218}{B}\;\right)}]$$

Notably, the *t*↓ and *t*_½_ values decrease with the increase in the AL, due to decreasing Factor ***A*** values, resulting from the increase in DL_CO_ and Va values (Table 1). The half-time calculated for AL = 1 (*t*_½_ = 305 min) is similar to the value stated in [1] for the half-time COHb removal at ambient environment.

### 2.7. Statistical Analysis

The model performance was evaluated by comparing the predicted COHb concentrations with the measured blood COHb values using least-squares regression. For each participant, two ALE values were determined: the ALE value derived directly from physiological parameters (*ALE_calc_*), and that yielded the best curve fit between predicted and observed COHb levels (*ALE_bf_*). Differences between the *ALE_calc_* and the *ALE_bf_* were analyzed across all participants to assess the model accuracy and interindividual variability. Notably, *ALE_calc_* and *ALE_bf_* are two variables derived from separate data streams, calculated against the measured values. Statistical analyses were performed using standard regression methods, with significance set at *p* < 0.05.

## 3. Results

### 3.1. Prediction of COHb Levels Using Subject-Specific Physiological Parameters

To evaluate the performance of the CFK model, we assessed its ability to predict blood COHb concentrations based on individually measured physiological and respiratory parameters. These predictions were then compared with the actual COHb values determined in the venous blood samples collected from the tested subjects. As indicated in Equation (3), the most influential variable in the CFK model is the ***b*** factor, which integrates diffusion capacity and ventilation data. For comparison, we also examined the MIL-STD-1472 model, which simplifies the CFK framework by substituting individual parameters with activity levels (AL). Since the two models are mathematically associated (Equation (7), and for every ***b*** factor an equivalent AL value could be calculated, allowing for alignment between the models on a continuous decimal scale, as shown in Table 3.

**Figure 2:**
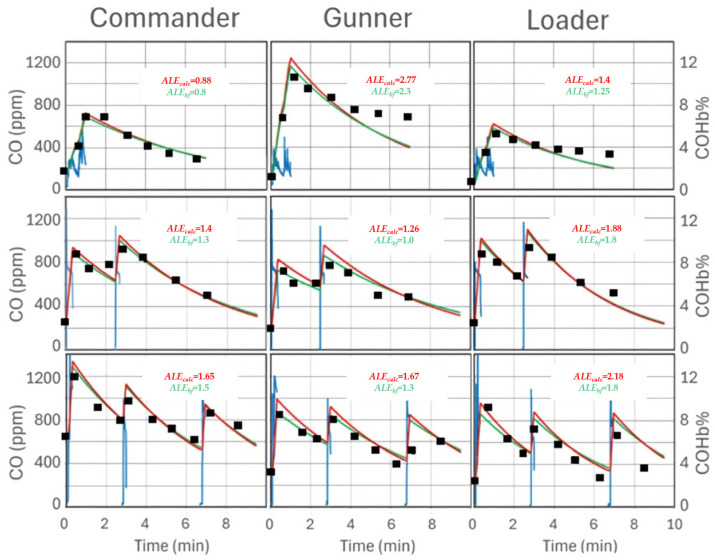
Typical COHb curves calculated based on *ALE_calc_* (red curves) and *ALE_bf_* (green curves) for three representative team members in each of Sessions 1, 2, and 3 (upper, middle, and lower panels, respectively). CO concentrations are shown in blue. COHb concentrations measured in the blood samples are shown as black squares.

**Figure 3:**
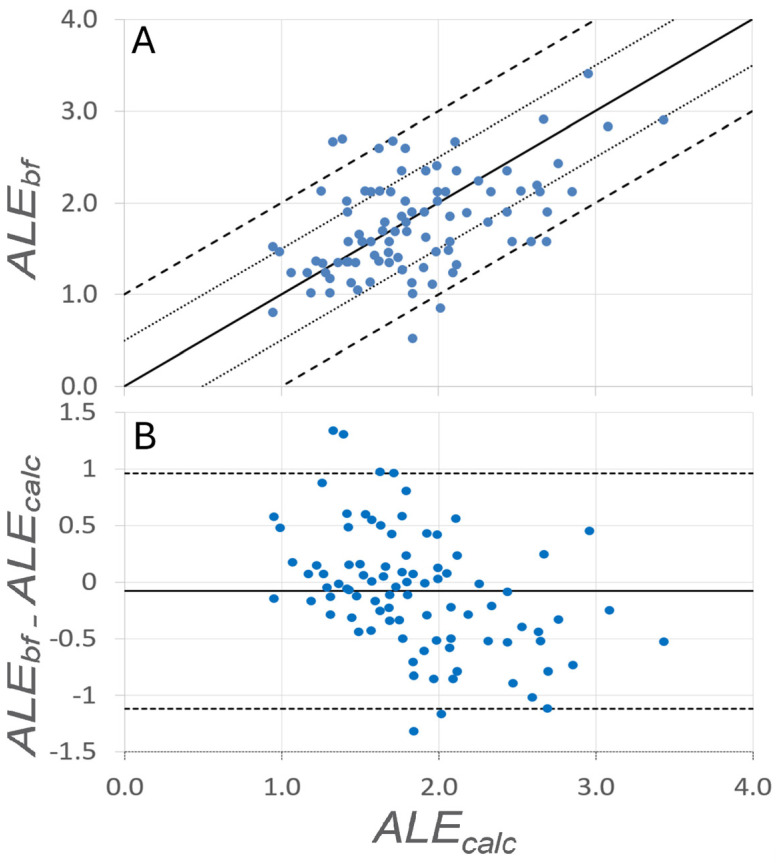
(**A**) Analysis of the *ALE_bf_* versus *ALE_calc_* for the full set of participants. The black solid line represents the equality line, and the dotted and dashed gray lines represent ±0.5 and ±1.0 units of deviation from equality, respectively. (RMSE = 0.56, r = 0.60, MPE = −0.008, PM20% = 0.53). (**B**) Bland–Altman analysis showing bias (solid line), 95% confidence intervals (CI), and limit of agreement (LoA) (dashed line).

Using a large group of volunteers who were exposed to CO generated during gunfire allowed us to determine the actual ALE values of the individual subjects, as described in Table 3. The values of the expected COHb% were calculated by the MIL-STD-1472 equation for each participant, based on the measured COHb_0_ and the calculated ALE (*ALE_calc_*). Separately, for each subject, the ALE value that yielded the best fit between the predicted and measured COHb trajectories was determined by least-squares regression (*ALE_bf_*). Representative examples for three participants from each firing session are shown in Figure 2, where the *ALE_calc_*-based curves (red) closely match the *ALE_bf_*-based fit (green). As shown in Figure 3, the ALE values did not exceed 3.0 in 98% of the tested participants, with an average and standard deviation of 1.82 ± 0.46, far below the AL = 4 suggested by the MIL-STD-1472 developers. Notably, 70% of the subjects showed a difference between the *ALE_calc_* and *ALE_bf_* of less than ±0.5, and 95% showed a difference of less than ±1.0.

These findings suggest that the conventional AL = 4 value recommended in the MIL-STD-1472 significantly overestimates COHb accumulation in most participants under these operational conditions. An adjustment to lower the AL values yields a more accurate and physiologically relevant estimation of CO toxicity risk during confined-space gunfire.

### 3.2. COHb Prediction Using the MIL-STD-1472 Model at Fixed Activity Levels

**Figure 4:**
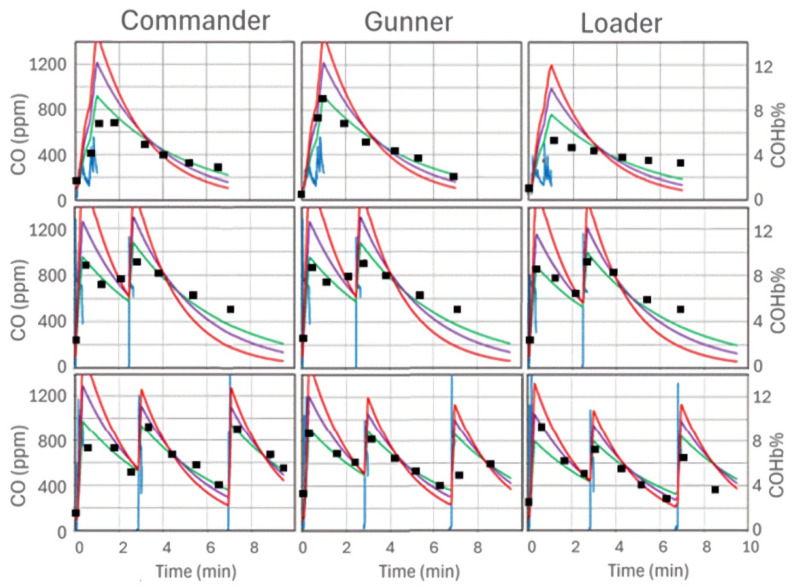
Typical COHb curves calculated according to MIL-STD-1472, based on AL = 2, 3, or 4 (green, purple, and red curves, respectively), for the same team members shown in Figure 2, representing Sessions 1, 2, and 3 (upper, middle, and lower panels, respectively). CO concentrations are shown in blue. Measured blood COHb concentrations in blood samples are shown as black squares.

**Figure 5:**
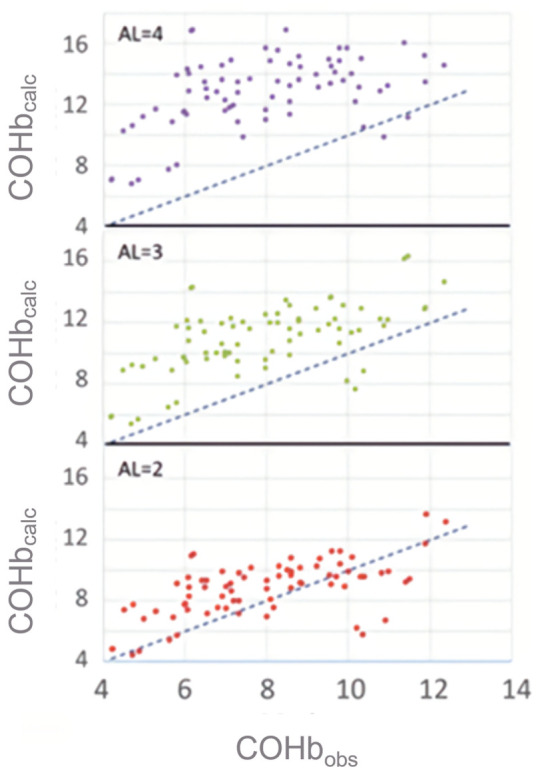
A comparative analysis of peak predicted COHb values versus the corresponding measured peaks in each participant. Model outputs are shown for AL = 2, 3, and 4 (green, purple, and red, respectively), while equality lines are indicated in orange (RMSE = 1.94, r = 0.61, MPE = 0.86, PM20% = 0.6, for AL = 2).

The measured blood COHb concentrations were next compared with the values predicted by the MIL-STD-1472 model using fixed activity levels of AL = 2, 3, or 4 (Table 1). In accordance with the assumptions of both the CFK and the MIL-STD-1472 formulations, a baseline COHb_0_ of 1% was applied. Because this assumption does not hold for heavy smokers [67,68], 18 participants with COHb_0_ > 3.5% were excluded from this analysis. The representative COHb time courses predicted using AL = 2, 3, and 4 are shown in Figure 4, alongside the measured blood COHb values. The peak predicted COHb concentrations for all participants were compared with the corresponding peaks measured in Figure 5. A strong correlation was observed between the measured COHb values and the MIL-STD-1472 predictions when AL was set to 2. Predictions using AL = 3 tended to modestly overestimate the measured COHb, whereas the use of AL = 4 consistently produced substantial overestimations across the participants.

A strong overall correlation was observed between the actual blood COHb values and those predicted by the MIL-STD-1472-based model when AL was set to 2 (Figure 5). Conversely, the use of AL = 4, as recommended in the original MIL-STD-1472, overestimated the COHb levels. These findings suggest that the more permissive AL = 2 or 3 provide a more realistic and operationally appropriate input for the MIL-STD-1472 model in the context of CO exposure during confined-space weapons firing.

### 3.3. Military Operational Implications of the MIL-STD-1472 Model

#### Simulation-Based Prediction of Firing Constraints

**Figure 6:**
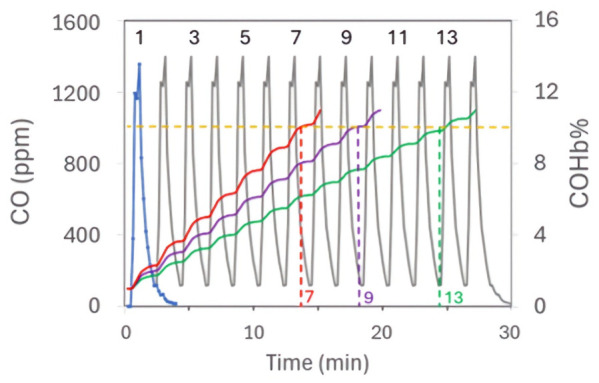
Simulated CO and COHb accumulation following repeated shell firings in a sealed AMV (gray lines). The simulation is based on extrapolation from a single-shell response (blue line), assuming firing every 2 min. The yellow dashed line represents the maximal COHb% allowed (10%). Colored curves represent predicted COHb levels at AL = 2 (green), AL = 3 (purple), and AL = 4 (red). Colored numbers at the bottom represent allowed repetitions for AL = 4, 3, and 2, respectively.

The MIL-STD-1472 model serves not only as a retrospective analytical tool but as a predictive framework for planning firing protocols under confined-space conditions. In operational settings, the model is routinely applied to simulate COHb accumulation resulting from repeated ammunition discharge, with the goal of avoiding physiologically hazardous thresholds. As illustrated in Figure 6, a common approach begins with an analysis of the COHb response to a single shell fired in a sealed AMV environment. This response curve serves as a baseline for computational modeling, typically implemented in spreadsheet-based tools, such as Excel, where the exposure is extrapolated to multiple firings at defined intervals. The simulation continues until the predicted COHb concentration reaches a critical limit, often set at 10%, beyond which cognitive and physiological impairment may occur [1].

The predicted number of allowable firings is extremely sensitive to the assumed activity level (AL). In the example shown, firing one shell every two minutes leads to substantial variation in the cumulative COHb, depending on the AL: at AL = 2, up to 12 firings may be permitted before reaching the 10% threshold, while at AL = 3, only 9 firings are allowed, and at AL = 4, the limit is reduced to 7 firings.

These findings emphasize the importance of accurate AL estimation in tactical planning and support the empirical observations that AL = 2–3 more closely reflects the metabolic workload of AMV crews during operational firing conditions.

## 4. Discussion

Carbon monoxide exposure results in the formation of COHb, which competes with oxygen for binding to Hb, thereby impairing oxygen transport and delivery to the tissues. Accordingly, the prediction of blood COHb concentrations serves as a surrogate marker for evaluating CO toxicity across various exposure scenarios, including military operations, mining, and firefighting environments.

The current study sought to compare the predictive validity of these models in a real-world military context. Because the present study was conducted within a military operational framework, the cohort consisted exclusively of young male soldiers aged 18–22 years. We acknowledge that this limits the direct generalizability of the findings to broader civilian populations, older individuals, females, or populations with significant medical comorbidities. At the same time, by utilizing a homogeneous group of one hundred trained crew members, matched in age, occupation, physical conditioning, and diet, we minimized the confounding variables inherent to general population-based applications of the CFK model.

To compare the results obtained with the CFK model, calculated from physiological and anthropometric data, with those expected using the MIL-STD-1472 model, we introduced the term AL equivalent (ALE), calculated using Equation (7). Based on the low workloads at which our experiments were conducted, under training conditions, the ALE values in the range of 1 to 3 were expected, and achieved, as shown in Figure 3 (1.82 ± 0.46). To ensure the integrity of the validation process, a strict mathematical separation was maintained between the predictive and optimization parameter: the *ALE_calc_* values were derived from the ***A*** and ***b*** factors which were calculated using anthropometric and physiological parameters, while the *ALE_bf_* value was calculated by the least-squares best fit method of blood COHb measured concentrations. Although the *ALE_bf_* value was obtained through the post hoc optimization of measured COHb trajectories, it was used as a diagnostic fitting parameter rather than as an independent validation variable. Although the two parameters, *ALE_calc_* and *ALE_bf_*, were calculated independently, the two ALEs were correlated well, as shown in Figure 3.

These results suggest that the MIL-STD-1472 model, which assumes AL = 4 for gun firing from AMVs, overestimates the COHb concentrations that accumulate in the blood during gun firing exposure to CO. Obviously, the use of AL = 4 leads to an over-estimation of the CO risk at gun firing. It is pertinent to note that higher deviations were observed in some participants, which occurred primarily during the later phases of prolonged exposure sessions. These discrepancies likely reflect cumulative physiological variability over time, including gradual changes in ventilation patterns, fatigue, or metabolic clearance, which are not fully captured by the static assumptions of the current models.

The next step was to evaluate the ability of the MIL-STD-1472 to predict the COHb concentrations in the blood of the soldiers using different AL settings (2, 3, or 4). As mentioned above, this model assumes COHb_0_ = 1%. Therefore, heavy smokers who had high values of COHb_0_ (>3.5) in their blood were excluded at this stage. As shown in Figure 3 and Figure 4, and in accordance with the results presented above, using AL = 4 results in an overestimation of the COHb values, while using AL = 2 provides a good fit between the predicted and the measured blood COHb values. With most individuals evaluated, using AL = 3 leads to predicted COHb values which are slightly higher than the actual blood values, and is therefore a recommended setting for the evaluation of CO toxicity when the physiological parameters of the tested individuals are not available. As shown in Figure 6, the value assigned to the AL directly affects the allowed number of repetitions of firing under certain conditions until a limit is approached.

It is pertinent to note that our experiments were conducted under training conditions that presume near-ambient O_2_ and limited CO_2_ accumulation. During combat, however, characterized by an elevated stress, and combined with a marked increase in heart and ventilation rates, the uptake of CO and the accumulation of blood COHb may be significantly higher, and AL = 4 may better represent the actual situation. In addition, it should be noted that extrapolation of the present findings to other occupational environments, such as mines, should be performed cautiously.

## 5. Conclusions

This study provides a field-based validation of the CFK and MIL-STD-1472 equations for predicting blood carboxyhemoglobin accumulation during confined-space military firing operations. Using continuous CO monitoring and serial blood COHb measurements obtained during live-fire exercises in armored motor vehicles, both models demonstrated reliable estimation of COHb formation under operational conditions characterized by near-ambient oxygen tension and limited CO_2_ accumulation.

The results indicate that the physiological workload experienced by AMV crew members during training exercises is substantially lower than the AL = 4 assumption currently recommended in the MIL-STD-1472 framework. The ALE analysis and direct comparison between the predicted and measured COHb concentrations showed that the AL values of 2–3 provide markedly more accurate predictions, whereas AL = 4 consistently overestimated the COHb accumulation and may therefore lead to unnecessarily restrictive operational limitations.

The ALE concept enabled quantitative alignment between the individualized CFK-derived physiological parameters and the workload-based MIL-STD-1472 model, providing a practical framework for comparing physiological and operational approaches to a CO exposure assessment. In addition, rearrangement of the MIL-STD-1472 equation enabled an estimation of the COHb recovery kinetics and post-exposure recovery times, parameters that may be important for operational planning.

Simulation analyses further demonstrated that relatively small differences in the AL selection substantially affect the predicted allowable firing rates before reaching the critical COHb thresholds, emphasizing the importance of realistic workload estimations.

Importantly, these findings primarily reflect controlled training conditions with relatively stable oxygen availability and limited CO_2_ accumulation. During actual combat conditions, increased stress, ventilation, and metabolic demand may significantly enhance CO uptake and COHb accumulation, potentially making higher AL assumptions more appropriate.

Overall, this work supports the refinement of MIL-STD-1472 activity assumptions for confined-space firing environments and may contribute to an improved CO exposure risk assessment, optimization of firing protocols, and safer operational planning in military and civilian settings involving intermittent high-level CO exposure.

## Figures and Tables

**Table 1:** The ***A***, ***b***, and ***B*** factors and the t½ values as functions of the AL.

AL	Effort Level	DL_CO_^1^	Va ^1^	*b* ^1^	*A* ^2^	*B* ^2^	t_½_^3^
		ml/min/mmHg	ml/min	mmHg-min/mL	min	1/mmHg	min
1	Sedentary	30	5000	0.17724	425	806	305
2		35	10,000	0.10053	241	1421	171
3	Light Work	40	15,000	0.07296	175	1958	123
4	Moderate Work	50	20,000	0.05596	134	2553	94
5	Hard Work	60	25,000	0.04544	109	3144	75

^1^ Data from [35,52]. ^2^ Data from MIL-STD-1472H [48]. ^3^ Calculated according to Equation (10), below.

**Table 2:** Calculation of physiological parameters.

Parameter	Formula	Ref
Body Surface Area (BSA)	0.007184 · W^0.425^ · H^0.725^	[64]
Dead Space Volume (Vd)	7.585 · H^2.363^/10000	[65]
Blood Volume (Vb)	3290 · BSA − 1229	[66]
Alveolar Ventilation Rate (Va)	(V_T_ − V_D_) · f.	

W and H are weight and height, respectively.

**Table 3:** Representative calculation of the ***b, A, B,*** and ALE values (Part 2) on the basis of the measured anthropometric and physiological parameters (Part 1) for three individual subjects as an example.

Part 1.											
No.	Age	Height	Weight	BSA	Vt	f	Vd	Va	HbO_2_	DLco	Vb
	A Years	H cm	W Kg	m^2^	mL	1/Min	mL	mL/min	mL/dL	mL/min/mmHg	mL
1	20	175	65	1.79	590	16.4	151	7192	20	42.08	4663
2	19	180	75	1.94	567	15.3	162	6193	20	44.38	5162
3	21	185	75	1.98	785	12.0	177	7348	20	46.02	5290
↓											
**Part 2.**											
**No.**			***A*** **^1^**		***B*** **^1^**			***B*** **^1^**		**ALE ^2^**	
			**Min**		**mmHg/mL/Min**			**1/mmHg**		**Min.**	
1			249		0.11092			1288		1.49	
2			303		0.12592			1134		1.27	
3			267		0.11278			1267		1.48	
↓											

^1^ ***A***, ***b***, and ***B*** factors are from Table 1. ^2^ ALE was calculated according to the data presented in Table 1. ↓: means that the calculations go on in the same way as with the other participants.

## Data Availability

The data supporting the findings of this study are available within the article. No additional datasets were generated or analyzed during the current study.
